# Three Research Strategies of Neuroscience and the Future of Legal Imaging Evidence

**DOI:** 10.3389/fnins.2018.00120

**Published:** 2018-03-01

**Authors:** Jinkwon Jun, Soyoung Yoo

**Affiliations:** ^1^KIAS Transdisciplinary Research Program, Korea Institute for Advanced Study, Seoul, South Korea; ^2^Human Research Protection Center, Asan Medical Center, Seoul, South Korea; ^3^Health Innovation Big Data Center, Asan Medical Center, Seoul, South Korea

**Keywords:** neuroscience and law, neurolaw, neuroethics, brain manipulation, brain function database

## Abstract

Neuroscientific imaging evidence (NIE) has become an integral part of the criminal justice system in the United States. However, in most legal cases, NIE is submitted and used only to mitigate penalties because the court does not recognize it as substantial evidence, considering its lack of reliability. Nevertheless, we here discuss how neuroscience is expected to improve the use of NIE in the legal system. For this purpose, we classified the efforts of neuroscientists into three research strategies: cognitive subtraction, the data-driven approach, and the brain-manipulation approach. Cognitive subtraction is outdated and problematic; consequently, the court deemed it to be an inadequate approach in terms of legal evidence in 2012. In contrast, the data-driven and brain manipulation approaches, which are state-of-the-art approaches, have overcome the limitations of cognitive subtraction. The data-driven approach brings data science into the field and is benefiting immensely from the development of research platforms that allow automatized collection, analysis, and sharing of data. This broadens the scale of imaging evidence. The brain-manipulation approach uses high-functioning tools that facilitate non-invasive and precise human brain manipulation. These two approaches are expected to have synergistic effects. Neuroscience has strived to improve the evidential reliability of NIE, with considerable success. With the support of cutting-edge technologies, and the progress of these approaches, the evidential status of NIE will be improved and NIE will become an increasingly important part of legal practice.

## Introduction: requirements for legal imaging evidence

Neuroscientific imaging evidence (NIE) has become an integral part of the criminal justice system in the United States. During 2005–2012, more than 1,500 judicial opinions have involved NIE (Presidential Commission for the Study of Bioethical Issues, 2015). However, in most legal cases, NIE is submitted and used only to mitigate penalties, because the court does not recognize it as substantial evidence (Salerno and Bottoms, 2009; Farahany, 2016). The incomplete acceptance of NIE is supported by criticisms regarding the validity of the imaging data (Aharoni et al., 2008; Pardo and Patterson, 2011; Jones et al., 2013; Buckholtz and Faigman, 2014; Dawid et al., 2014; Faigman et al., 2014), which have raised concerns regarding the evidential reliability of imaging evidence.

For example, in *People v. Ruiz*, the accused claimed incompetence to stand trial on the basis of neuropsychological testimony^1^. Two experts examined Ruiz's brain and diagnosed him with a severe language disorder. Their opinion was based on neurobiological evidence supporting poor development of the left part of the brain, which is known to be associated with language skills. The trial judge concluded that the evidence supported the incompetence of the accused. This legal decision was based on the argument shown below.

Premise #1: In the present case, the defendant has a deficit in brain area B.
Premise #2: In other studies, brain area B is putatively responsible for cognitive process M.
Conclusion: Thus, the damage in area B of the brain of the present case demonstrates his incompetence in cognitive process M.

Premise #1 is testable by investigation of the culprit; therefore, premise #2 is the key for establishing the validity of the conclusion. To prove premise #2, neuroscience should demonstrate that brain area B is indeed putatively responsible for cognitive process M. However, it is difficult to find a clear relationship between mental processes and brain regions. Critics have emphasized that there are numerous situations wherein premise #2 can be discredited, such as degeneration (Friston et al., 2006) and modulation (Logothetis et al., 2001). Therefore, critics claim that most neuroimaging studies show only correlations, rather than causations (Aharoni et al., 2008; Buckholtz and Faigman, 2014). Moreover, they claim that neuroscience is incapable of confirming the role of a particular brain region in an individual act (Dawid et al., 2014; Faigman et al., 2014).

However, such arguments are misguided, because the law does not require complete proof of a causal relationship. The federal rule of evidence 401 defines the Test for Relevant Evidence as follows.

*Evidence is relevant if*

(a) *it has any tendency to make a fact more or less probable than it would be without the evidence*

Furthermore, the federal rule of evidence 702 defines the condition for Testimony by Expert Witness as follows:

(b) *the testimony is based on sufficient facts or data;*
(c) *the testimony is the product of reliable principles and methods; and*
(d) *the expert has reliably applied the principles and methods to the facts of the case*.

According to the federal rules of evidence, if sufficient data are gathered in accordance with reliable principles and methods, NIE can be considered significant evidence. The court is not concerned about the evidence being complete or incomplete. Accordingly, the degree of reliability of NIE is the actual issue.

From this perspective, the remarkable improvements in the field of neuroscience in recent years should be given importance. Newly developed techniques and theoretical tools have contributed to an improvement in the evidential reliability of imaging data. Accordingly, the aim of this article is to explain how these developments are overcoming the reliability issues of NIE. We have classified the efforts of neuroscientists into three research strategies: cognitive subtraction, the data-driven approach, and the brain-manipulation approach. All strategies share the same goal of confirming whether brain area B putatively engages in cognitive process M (hereafter referred to as B→M)^2^, which will consequently improve the reliability of premise #2. The three approaches are presented in Table 1.

**Table 1:** Research strategies for B→M.

**Strategy**	**Method**
Cognitive Subtraction	Manipulation of cognitive process M and observation of brain area B
Data-Driven Approach	Statistical processing of data obtained from cognitive subtraction
Brain-Manipulation Approach	Manipulation of brain area B and observation of cognitive process M

The first part of the article explains cognitive subtraction, which is the most common strategy in the field. However, it has limitations with regard to legal use, as pointed out by critics. Then, we introduce the data-driven and brain-manipulation approaches as more appropriate alternatives in the subsequent two sections. The data-driven approach brings data science into the field of imaging neuroscience. The brain-manipulation approach is based on novel methodologies for controlling the brain. In the second and third sections, we discuss how the data-driven and brain-manipulation approaches, respectively, can increase the reliability of NIE with the support of cutting-edge technologies. We conclude by asserting that criticisms against the legal use of NIE may no longer be appropriate because of advances in neuroscience.

## Cognitive subtraction and its limitations

Most experimental designs in neuroscience research follow the following format. First, researchers use imaging technology, such as functional magnetic resonance imaging (fMRI) or positron emission tomography (PET), to scan brain activation patterns (such as the blood oxygen level-dependent [BOLD] signal) in subjects while they are not engaged in any kind of task. This is referred to as the resting state or baseline. The researchers then scan the brain while the subjects perform a cognitive task, such as face recognition, memory loading, or auditory stimulation. We can let C0 be the resting state condition and C1 be the condition where the brain is engaged in a psychological process of interest (mental process M). The difference in the BOLD signal between C0 and C1 in brain region B is the measure of interest. This method for evaluating the relationship between brain and function is known as cognitive subtraction (Friston et al., 1996; Logothetis et al., 2001).

**Figure 1:**
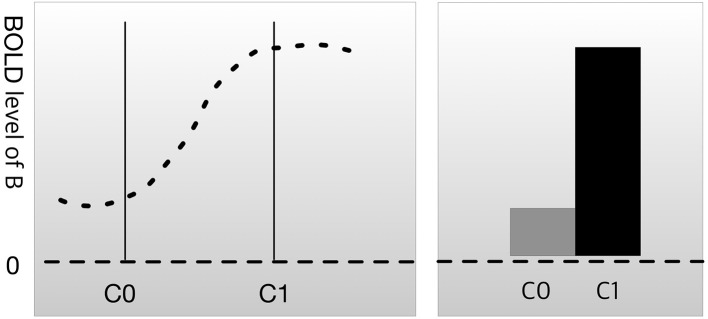
Cognitive subtraction. C0 is a control condition, and C1 is an experimental condition. The height of the graph represents the level of the blood oxygen level-dependent (BOLD) signal in brain region B. The difference in the BOLD signal between C0 and C1 is considered to indicate that brain region B is engaged in C1.

In Figure 1, the result of cognitive subtraction is interpreted as R1 engaging in a cognitive process related to C1, because the BOLD signal in R1 is significantly increased during C1 compared with that during C0. However, cognitive subtraction alone is not sufficient to confirm inference B→M, because cognitive subtraction merely shows that brain area B is activated (inferentially, M→B) during mental process M. There are conditions that render M→B insufficient as evidence for B→M.

**Figure 2:**
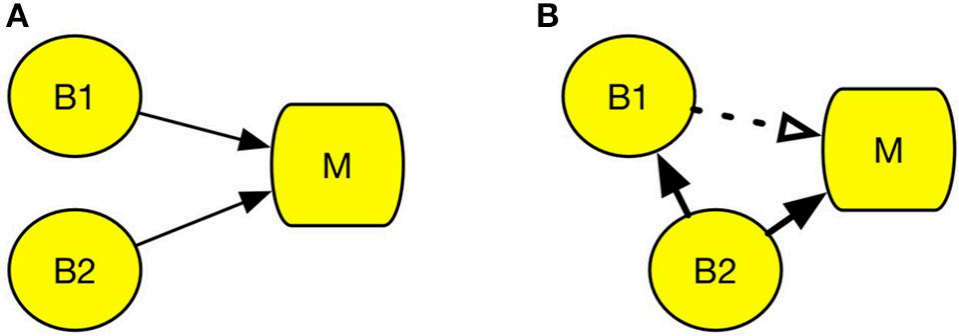
Conditions that undermine cognitive subtraction. **(A)** Degeneracy. **(B)** Correlation. Circle B refers to a specific brain region and square M refers to a specific psychological process. The solid line arrow indicates a causal relation and the dotted line arrow indicates a correlation.

Figure 2A represents a case of degeneracy, wherein multiple brain regions engage in the same psychological process (Noppeney et al., 2004). The left parietal cortex and left putamen are examples of degeneracy, because both regions can be engaged in the cognitive process of reading, and the region activated during reading differs across individuals (Seghier et al., 2008). Therefore, it is difficult to determine the region responsible for reading using cognitive subtraction only. An example of correlation, which is illustrated by the dotted line in Figure 2B, could be neuromodulation, which is an activation that facilitates control of other brain regions. In Figure 2B, while activation of B2 is the actual cause of M, activation of B1 is a mere response to the change in B2. According to Logothetis (2008), the majority of neurons do not deliver a signal; instead, they modulate the activity of other neurons. This may lead to an incorrect interpretation that B1 is the area responsive to experimental stimuli. However, when B1 is damaged, process M remains intact.

**Figure 3:**
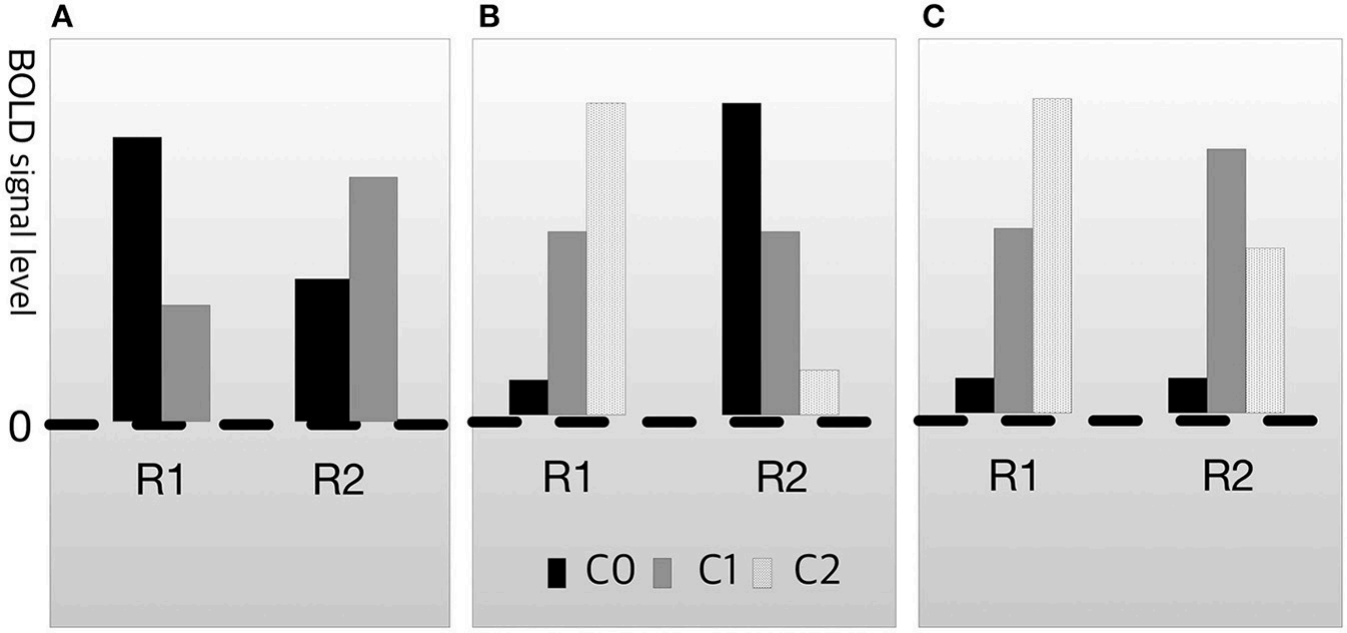
Types of dissociation. **(A)** Double dissociation. **(B)** Crossover double association. **(C)** Reverse associateon.

In both cases, cognitive subtraction can fallaciously judge that B1 is engaged in process M. However, neuroscientists are aware of this problem and have also attempted to resolve it. The conventional manner of dealing with this issue is known as dissociation (Teuber, 1955; Shallice, 1988). Dissociation methods aim to ascertain whether two brain regions are underwritten by the same process (Henson, 2005, 2006a,b). The basic logic of dissociation is to exclude fallacious possibilities, such as those described for the cases in Figure 2, using additional experimental data. For example, assume that C0 is the condition-related mental function F0 and C1 is the condition-related function F1. When the activation pattern of R1 and R2 on F1 and F2 is as shown in Figure 3A, the relationship between R1 and R2 is called double dissociation (Henson, 2005). If R1 is only engaged in F0 and R2 is only engaged in F1, there is evidence indicating that the two regions are not engaged in the same job. Based on this logic, researchers can separate the brain area with the function from the irrelevant areas. However, double dissociation is not sufficient to draw the conclusion that B→M because it cannot exclude the possibility that R1 and R2 are antagonistic. To resolve this, at least three experimental conditions are required. Let C0 be the control condition, C1 be the condition that is less engaged in cognitive process M, and C2 be the condition that is more engaged in function F (i.e., assuming a linearity among C0, C1, and C2). Then, if the activation patterns are as shown in Figure 3C, which are collectively called reverse association (Dunn and Kirsner, 1988; Henson, 2005; Machery, 2012, 2014), R1 and R2 are not antagonistic. If they suppress each other, the result would be as shown in Figure 3B, not as shown in Figure 3C.

In this manner, neuroscientists can improve the reliability of imaging data by accumulating experimental data from various conditions. This research strategy, often referred to as imaging dissociation (Machery, 2012), is the basic strategy used to elucidate the relationship between the brain area and cognitive function. However, it should be noted that this approach also cannot provide a complete confirmation of B→M (Friston et al., 2006; Poldrack, 2006; Logothetis, 2008; Anderson, 2010; Sternberg, 2011). The ultimate reason is that the human brain is complex; therefore, there are near-infinite conditions for confirming B→M, and testing of all conditions is extremely demanding (Aguirre et al., 2003).

Nevertheless, scientific evidence and other evidence do not have to be perfectly confirmed in order to have legal reliability. For example, DNA fingerprinting is now widely accepted as substantial evidence, even though it is not incontrovertible. When DNA evidence is properly collected and analyzed by qualified experts, its credibility is admitted by the court (National Research Council, 2011). The same principle should be applied to neuroscientific evidence.

The Daubert standard, which has become the benchmark test for the reliability of neuroscientific evidence, has set the conditions for the legal use of NIE as follows:

1. whether the theory or technique can be tested and has been tested
2. whether the theory or technique has been subjected to peer review and publication
3. the known or potential rate of error of the method used and the existence and maintenance of standards controlling the technique's operation
4. whether the theory or method has been generally accepted by the scientific community^3^.

In 2012, in *United States v. Semrau*, the Supreme Court decided that NIE for lie detection, which is mainly based on cognitive subtraction, satisfies conditions (1) and (2), but not conditions (3) and (4)^4^.

As the sentence shows, NIE has not reached the level achieved by DNA evidence at present. However, the decision left a caveat for the future.

*In the future, should fMRI-based lie detection undergo further testing, development, and peer review, improve upon standards controlling the technique's operation, and gain acceptance by the scientific community for use in the real world, this methodology may be found to be admissible even if the error rate is not able to be quantified in a real world setting*^4^.

As stated above, if neuroscience research succeeds in improving the reliability of NIE, it will be accepted as substantial evidence. It is thus important to understand the problems involved in cognitive subtraction. The first limitation is related to the issue of causality. In a normal experimental setting, brain area B is the cause and cognitive process M is the effect. This indicates that cognitive subtraction is a method for manipulating the effect and observing the cause. Generally, discerning the cause from the effect is difficult, particularly in cases involving a complex structure. Second, establishment of a reliable conclusion regarding B→M via cognitive subtraction is a very demanding process, because the human brain is complex and much information is required to prove that B→M.

Fortunately, the two major drawbacks of cognitive subtraction are being resolved because of advances in the field of neuroscience, which can be characterized by two trends: the data-driven approach and the brain-manipulation approach. These two strategies illustrate how neuroscientists have attempted to deal with the reliability issue pertaining to imaging evidence for B→M. In the next two sections, we introduce and describe state-of-the-art scientific progress in these areas and discuss how it can shed light on the future of NIE.

## Data-driven approach

As discussed above, cognitive subtraction is not a suitable strategy for obtaining legal imaging evidence. One problem is that the collection of brain–function data via dissociation is a very demanding and labor-intensive process. However, the data-driven approach tackles this issue from a different angle with the help of data science. The core concept of the data-driven approach is that a sufficiently large amount of information can substantially increase the reliability of the brain–function relationship. Accordingly, this approach utilizes large databases and computerized data processing (Yarkoni et al., 2011; Hutzler, 2014; Poldrack and Yarkoni, 2016), thus justifying its name.

The basic logic of the data-driven approach can be summarized as follows.

*If a region is active across more psychological functions, it will provide less support for* B→M.
*If a region is active across fewer psychological functions, it will provide more support for* B→M.

The idea is simple. If a region is engaged in only one mental process, its activation would provide good evidence for the occurrence of this mental process. However, if the region is engaged in various mental processes, then its activation would not yield good evidence for the occurrence of the mental process. Among the various implements for this idea, we will focus on the Bayesian approach^5^ which is based on the use of the Bayesian rule to represent and calculate the reliability of evidential support:

(1) $$\begin{array}{r} P\left(M|B\right)=\frac{P\left(B|M\right)P\left(M\right)}{P\left(B|M\right)P\left(M\right)+P\left(B|\sim M\right)P\left(\sim M\right)} \end{array}$$

^6^ Bayesian rule for B→M

Equation 1 is the Bayesian rule for measuring the certainty of regional data obtained using imaging technology (Poldrack, 2006; Yarkoni et al., 2011; Rubin et al., 2016). M refers to the ongoing mental process and B refers to activation of the region of interest. *P*(*M*|*B*) is the conditional probability of occurrence of psychological process M on activation of brain region B, which is interpreted as the degree of credibility of the inference B→M. *P*(*M*) is the base rate for mental process M, *P*(*B*|*M*) is the conditional probability of activation of brain region B on occurrence of psychological process M, and *P*(*B*|~*M*) is the conditional probability of activation of region B without the occurrence of psychological process M. The usefulness of the Bayesian rule is that it provides an update on the present degree of reliability from new evidence. When a task relevant to M induces activation of A, *P*(*B*|*M*) should increase. Conversely, when a task that is irrelevant to M induces activation of A, *P*(*B*|~!*M*) should increase. If *P*(*B*|*M*) increases, then *P*(*M*|*B*) also increases; in contrast, if *P*(*B*|~!*M*) increases, then *P*(*M*|*B*) decreases. In particular, it is important that *P*(*B*|*M*) and *P*(*B*|~!*M*) data can be obtained via cognitive subtraction.

In this way, the Bayesian approach can mediate data obtained from cognitive subtraction to B→M. However, even though this approach works well, it is impractical to use a single study to compare the various conditions for adequate justification of B→M. Meta-analyses, which depend on manpower, cannot avoid the scalability problem, because humans can only process a limited amount of data. However, the data-driven approach, which utilizes data science technology, such as machine learning and artificial intelligence, is not limited by the information processing capacity. Recently, studies in neuroscience established brain–function databases, such as BrainMap (Laird et al., 2005), Brede (Nielsen et al., 2004), SuMS (Dickson et al., 2001), OpenfMRI (Poldrack, 2011), NeuroVault (Gorgolewski et al., 2015). All these are automated platforms for data collection, storage, and analysis. For example, Neurosynth (http://www.neurosynth.org) uses data mining technology and a computational linguistic method for the automatic extraction of information from published articles and reports, ultimately generating clear, understandable images from the gathered information (Yarkoni et al., 2011). Currently, the Neurosynth database contains more than 10,000 articles and 36,000 discrete activation patterns. Furthermore, brain imaging standards such as the Neuroimaging Data Model (http://www.nidm.nidash.org) and the Nipype (Gorgolewski et al., 2011) are being developed; these will provide fully automatic, reproducible, shareable, and open-source analysis pipelines. With large databases such as these, neuroscientists are now trying to expand research in imaging science from region-level studies to whole brain-level studies (Del Pinal and Nathan, 2017). This approach is aiding in determination of the relationships among multiple brain regions and cognitive function in order to overcome the issue of the lack of specificity (Nathan and Del Pinal, 2017).

With regard to the legal context for NIE, it is important that these databases and analysis programs provide a criterion for evaluating the strength of B→M. Bayesian statistics conventionally use the Bayes factor for evaluating the conclusion. Table 2 shows an example of the assessment criteria (Kass and Raftery, 1995; Jeffreys, 1998).

**Table 2:** Assessment table for the Bayes factor.

**Value of the Bayes factor**	**Reliability of the inference**
1–3.2	Not worth more than a bare mention
3.2–10	Substantial
10–100	Strong
>100	Decisive

In case of Equation (1), the Bayes factor is $\frac{P\left(A|M\right)}{P\left(A|\sim M\right)}$, and the value of this formula is understood as the reliability of the imaging evidence. For example, Poldrack (2006) tested whether activation of Broca's area is engaged in language function using the BrainMap database. They derived a Bayes factor of 2.3, which was below 3.2 and consequently considered to represent weak evidence. In contrast, the ventral striatum data from the study by Ariely and Berns (2010) had a Bayes factor of 9, which was considered to represent moderately strong evidence.

In this manner, the data-driven approach can provide an error estimation for B→M. It will grade the evidence for the degeneration case in Figure 2A as untrustworthy, because M was associated with B1 in some cases and B2 in some. It provides an important advantage for NIE, because the Daubert standard requires estimation of the error rate.

*The known or potential rate of error of the method used and the existence and maintenance of standards controlling the technique's operation*^3^.

The data-driven approach can compensate for this limitation of NIE. Accordingly, a large brain–function database will lead to progress in the legal use of NIE. Moreover, the technology for data storage and analysis accelerates progress, thereby leveraging the scale and breadth of imaging data and increasing the power of the data-driven approach with the passage of time.

## Brain-manipulation approach

The issue of causality with NIE is commonly pointed out by critics (Feigenson, 2007; Aharoni et al., 2008). However, the brain-manipulation approach is a crucial advancement for resolution of this problem. Conventionally, the basic idea of causality in science is paralleled by intervention of the antecedents (Woodward, 2005). For imaging science, the antecedent is the brain area. As discussed before, cognitive subtraction merely controls the consequence (i.e., the cognitive process), which is the reason for it being insufficient to confirm causality.

If the activation of a specific brain region can be freely manipulated, direct testing of B→M becomes possible. However, conventional brain-manipulation methodologies have at least two major obstacles. First, they are mostly invasive, indicating that they can result in irreversible damage to the subject. Because there is no scientific consensus that animals can perform moral or ethical cognitive processes, legal imaging evidence should be collected from human subjects. Although various brain manipulation techniques are available, most of them require cranial surgery or may possibly damage the brain tissues. Therefore, such methods cannot be applied to humans for ethical reasons. Second, conventional methods are associated with a low spatiotemporal resolution. NIE for legal issues have highly sophisticated functions such as moral decision making, social relationships, and impulse control, among others. Because these cognitive processes are implemented by complex neuronal networks, accurate manipulation methods are necessary. For example, in lesional studies employing a conventional manipulation method, a patient with brain damage is compared to a healthy individual. Generally, a lesion includes a large number of neurons; therefore, mental processes cannot be accurately distinguished.

However, the field of neuroscience has developed safe and precise methods for brain manipulation. The brain-manipulation approach depends on state-of-the-art technologies, such as electric current stimulation, transcranial magnetic stimulation (TMS), optogenetics, and ultrasound, among others. Electric current stimulation, which uses a weak electric current for activation or deactivation of neurons, has several variations. Implanted microelectrode arrays or deep brain stimulation (DBS) utilize a chip of electrodes to stimulate a certain brain region. DBS is not only recognized as a safe instrument, but is also widely used for the treatment of Alzheimer's disease or treatment-resistant depression (Benabid et al., 2009). It can also stimulate subcortical regions. However, implantation methods still require head surgery. In contrast, transcranial direct current stimulation (tDCS) and its variations (transcranial alternating current stimulation, transcranial random noise stimulation, etc.) do not require cranial surgery. For typical tDCS, a large pad (~25 cm) is attached to the skin on the head for delivery of an electrical current. High-definition tDCS, which was recently developed (Nitsche et al., 2008; Caparelli-Daquer et al., 2012), provides much better spatial resolution of ~1 cm^2^. TMS is expensive and requires a trained technician; however, it provides better spatiotemporal resolution than tDCS. Instead of direct delivery of the current, TMS uses electromagnetic induction. The coil, which is placed near the head of the subject, generates an electromagnetic field and produces a weak current in the target region of the brain. High-definition TMS provides a spatial resolution of ~0.5–1 cm^2^ (Sliwinska et al., 2014), whereas newly developed micromagnetic stimulation coils provide a spatial resolution of ~500 μm (Bonmassar et al., 2012).

Other cutting-edge techniques are also overcoming the limitations of the conventional methods (Lewis et al., 2016). Optogenetics uses genetically modified neurons that express light-sensitive channel proteins on their membrane; therefore, researchers can control their activation or deactivation using light. It provides a surprisingly high spatiotemporal resolution (Aston-Jones and Deisseroth, 2013; Häusser, 2014; Adamantidis et al., 2015; Deisseroth, 2015). Recently developed organic light-emitting diode arrays have 6 × 9-μm^2^ elements, which are smaller than a neuron (Steude et al., 2016). However, there is an important hurdle for application of this method to humans, because optogenetics requires genetic modification. However, viral vectors are known to be relatively safe for consideration in clinical trials (Gilbert et al., 2014). Noninvasive delivery methods are also under development (Wang et al., 2017). Ultrasound methods thus have the advantage of being noninvasive. They use a mechanical pressure wave (sound wave) with a high frequency (>20 kHz), which can be transmitted through solid structures, including bone and soft tissues. Intensive ultrasound (over 1 w/cm^2^) controls neuronal excitation by producing thermal effects (Tufail et al., 2010, 2011). Studies on ultrasound techniques have reported spatial resolutions < 3mm^2^. Researchers are aiming to select brain regions < 1mm^2^, which is five times better resolution than that achieved by TMS. Another method attracting attention is temporal interference (TI) stimulation (Grossman et al., 2017). In this approach, researchers use two pairs of surface electrodes to generate 2 and 2.01-kHz sinusoidal stimulations concurrently, and the envelope of the two electrical stimulations results in a 10-Hz beat frequency in the deep brain region. While a neuron does not respond to a high-frequency stimulation, it does respond to a low-frequency stimulation. Using this feature, TI manipulates the deep brain region in a noninvasive manner. Neuroscientists are now developing a TI stimulation approach that uses multiple electrodes in order to expand its resolution. Many have deemed this technology as a breakthrough in the field of neuronal modulation (Dmochowski and Bikson, 2017).

We summarized the brain manipulation technologies in Table 3. The future of the brain-manipulation approach looks promising. It cannot be said that these methods have completely conquered the long-standing obstacles for brain control, but they are rapidly improving and neuroscientists and engineers are continuing to invent new technology. Neuroscience has already applied manipulation methods with imaging techniques (Wagner et al., 2007; Bestmann and Feredoes, 2013), for example, TMS with fMRI (Bestmann et al., 2008; Ruff et al., 2009; Siebner et al., 2009) and optogenetics with fMRI (Lin et al., 2016). With regard to methodological improvement, the manipulation approach is expected to be useful for solving the issue of causality (Dijkstra and de Bruin, 2016). For example, manipulation tests can rule out the correlational error in Figure 2B. If we manipulate B1, then no change will be observed in M; thus, we can conclude that B1→M is a mere correlation. Henson mentioned that “*imaging data from an experimental manipulation … are no more correlational”* (Henson, 2005, p. 222), indicating that the results obtained from manipulation will be much more reliable than correlations.

**Table 3:** Brain manipulation methods.

**Method**	**Non-invasiveness**	**Spatial resolution**
Legion study	Yes	Very low
Deep brain stimulation	No	Low (can stimulate deep brain areas)
Transcranial direct current stimulation	Yes	<1 cm^2^
Transcranial Magnetic Stimulation	Yes	0.5–1 cm^2^
Optogenetics	No	Very high
Ultrasound	Yes	1–3 mm^2^
TI (temporal interference) stimulation	Yes	Low (can stimulate deep brain areas)

In addition, manipulation methods can complement the data-driven approach. For example, dynamic causal models (DCMs) are used to test the interaction of TMS-induced neuronal changes with cognition (Esser et al., 2005; Cona et al., 2011). A neural model built by DCMs predicts the impact of physiological signals on cognition, and researchers can manipulate the brain region to test its prediction. This illustrates how manipulation and neuroimaging can be used together. Confirmation of results obtained using the data-driven approach with the manipulation approach will further increase the reliability of NIE. Thus, these two research strategies can exhibit synergistic effects.

## Conclusion: development of neuroscience and future of legal NIE

We described three neuroimaging strategies for legal use. The first strategy, cognitive subtraction, is a relatively old and problematic approach and is considered unreliable. The other two approaches, the data-driven and brain-manipulation approaches, are rapidly overcoming the limitations of cognitive subtraction. Critics assert that there is a significant gap between NIE and legal evidence, and that neuroscience can only demonstrate correlations (Aharoni et al., 2008). Furthermore, these two types of evidence have different purposes (Dawid et al., 2014) and are consequently incommensurable (Buckholtz and Faigman, 2014). However, in the present article, we have shown that neuroscience and the law of evidence pursue the same goal, which is to confirm the evidential reliability of NIE. Cutting-edge technologies increase the possibility of achieving this common goal. We therefore argue that the gap pointed out by critics is being narrowed by the efforts of neuroscientists.

We are convinced that both approaches will be applied in legal practice in the near future. For example, Gur and his colleagues at the University of Pennsylvania established a standard procedure for the legal use of NIE, which is called the Neuroforensics Service (Gur et al., 2016). They constructed the computerized neurocognitive battery (CNB) for analyzing the brain–function relationship (Gur et al., 2010). We want to emphasize that CNB can be significantly improved by application of both aforementioned approaches. First, because it is not an automated database, it may significantly benefit from the data-driven approach. A large brain–function database built with data technology can provide a wider range of data and better error estimates. Second, the manipulation method can provide an effective re-examination of known data, thereby making CNB more trustworthy. Furthermore, in cases where no data are available, a manipulation experiment can provide an appropriate solution.

Therefore, the two trends in neuroscience development are likely to make the legal use of NIE more reliable. Neuroscientists have striven to improve the evidential reliability of NIE and have achieved considerable success. As their research progresses, NIE will become an increasingly important part of legal practice.

## Author contributions

JJ provided the core idea and wrote the draft of the article. SY developed the idea, provided the summary of technological details, and approved the final version of the manuscript.

### Conflict of interest statement

The authors declare that the research was conducted in the absence of any commercial or financial relationships that could be construed as a potential conflict of interest.

## Footnotes

^1^People v. Ruiz. F057116. (Cal: Court of Appeal, 5th Dist., 2010).

^2^B→M is often referred as “reverse inference.” This, however, is not only a broader concept than B→M, but is also very technical; therefore, we intentionally avoid the use of this term. See Huettel et al. (2009).

^3^Daubert v. Merrell Dow Pharmaceuticals 509 U.S. 579 (1993). p. 593-594.

^4^United States v. Semrau, 693 F.3d 510 (6th Cir. 2012), p. 31.

^5^The likelihood approach may be another influential method. See Machery (2014).

^6^This equation is adopted from Poldrack (2006).
